# Estimating additional schooling and lifetime earning obtained from improved linear growth in low- and middle-income countries using the Lives Saved Tool (LiST)

**DOI:** 10.7189/jogh.12.08004

**Published:** 2022-04-02

**Authors:** Hannah Tong, Christopher G Kemp, Neff Walker

**Affiliations:** Department of International Health, Bloomberg School of Public Health, Johns Hopkins University, Baltimore, Maryland, USA

## Abstract

**Background:**

Policymakers seeking to prioritize the use of restricted financial resources need to understand the relative costs and benefits of interventions for improving nutritional status. Improved linear growth can lead to increased education attainment and improved economic productivity in low- and middle-income countries (LMICs), though these non-health-related benefits are not reflected in current long-term modelling efforts, including the Lives Saved Tool (LiST). Our objective was to integrate the effects of improved linear growth on non-health related benefit into LiST by estimating subsequent gains in years of schooling and wage earnings. We then estimated the impacts of reaching the Sustainable Development Goals (SDGs) target for stunting in South Asian countries on lifetime productivity.

**Methods:**

In the first step, we used LiST outputs to estimate the improved linear growth due to scaled-up nutrition interventions and used published estimates to quantify the education gain resulting from an increase in height for age z-score (HAZ). In the second step, we used published country-level estimates on economic returns to schooling to quantify the relative gains in wages that children born today will experience because of their additional education attainment in the future. In the last step, we used country-level data on wages to estimate the net present value of future earnings gained due to early childhood growth improvement per birth cohort.

**Results:**

If South Asia countries reach the SDG target by 2025, an estimated 8.6 million years of schooling will be obtained by six birth cohorts of 2020 to 2025. These six birth cohorts will also gain an estimated US$64 893 million in the present value term, at a 5% discount rate, in lifetime earnings. India has the largest expected gain in years of schooling (7367 years) and lifetime earnings (US$59 390 million in present value terms, at a 5% discount rate).

**Conclusions:**

Two non-health-related benefits of improved linear growth – additional years of schooling and lifetime earnings – are added in LiST. Together with LiST costing, users can now conduct both cost-effective and benefit-cost analyses. Using both analyses will provide more comprehensive insights into nutrition interventions' relative costs and benefits.

Essential nutrition interventions that address the immediate and underlying determinants of fetal and child nutrition and development offer a diverse range of health-related and non-health-related benefits, including increasing lifetime productivity and preventing mortality and morbidity [1,2]. Policymakers need to understand each intervention’s relative costs and benefits to prioritize nutrition interventions for scale-up, especially in low- and middle-income countries with severely restricted financial resources. Benefit-cost analysis (BCA) is a form of economic evaluation in which the measured health and non-health-related effects of interventions are monetized, aggregated, and compared to the full cost of intervention implementation [3]. BCA can help decision-makers maximize health and economic gains, given the limited resources. Properly allocated resources will also promote the achievement of the Sustainable Development Goals (SDGs), particularly SDG 2: End hunger, achieve food security and improved nutrition, and promote sustainable agriculture [4].

The Lives Saved Tool (LiST) includes a comprehensive list of nutrition and health interventions, and models the impact of increasing coverage of these interventions on maternal, neonatal, and child health (MNCH) and child growth, including both wasting and stunting in children [5]. LiST also includes a costing module that estimates costs-to-scale of each intervention, disaggregated into drug and supply costs, labor costs, other recurrent costs, capital costs, and above-service delivery costs [6]. Overall, LiST provides a user-friendly tool to estimate both the full financial costs of scaling up interventions and the resulting impact on maternal and child health. Currently, LiST only models the effects of stunting reduction on subsequent population mortality and morbidity. It does not estimate the non-health benefits of reduced stunting, though there is strong evidence of an association between improved linear growth and additional years of schooling and improved economic productivity in LMICs [7-9]. This limits the utility of LiST for the BCA of nutrition interventions. Our objective was to integrate the effects of improved linear growth on lifetime economic productivity into LiST by estimating subsequent gains in years of schooling and wage earnings. We present our methods and estimate the impact of reaching the SDG target for stunting in South Asian countries on lifetime productivity.

## METHODS

### Overview

**Figure 1:**
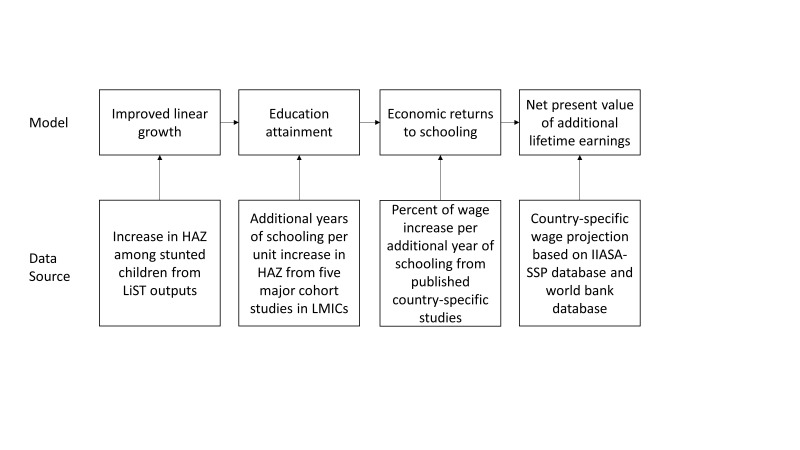
Conceptual model and data sources. HAZ – Height-for-age z-score.

We used the work of Fink et al. to estimate the association between improved linear growth and economic outcomes [10]. The method involved three steps: in the first step, we used LiST outputs to estimate the improved linear growth due to scaled-up nutrition interventions, and we used published estimates to quantify the education gain resulting from an increase in height for age z-score (HAZ). In the second step, we used published country-level estimates on economic returns to schooling to quantify the relative gains in wages that children born today will experience because of their additional education attainment in the future. In the last step, we used country-level data on wages to estimate the net present value of future earnings gained due to early childhood growth improvement per birth cohort. **Figure 1** summarizes the conceptual model and the data source used in each step.

### Improved linear growth

In LiST, country-specific data on stunting prevalence come from nationally representative household surveys. The survey data were re-analyzed to produce estimates of stunting in 4 categories (>-1, -1 to -2, -2 to -3, and<-3 z scores) on the basis of the more recent WHO growth standards for each of 5 age groups (<1, 1-5, 6-11, 12-24, and 24-59 months) [11]. From the LiST outputs, we estimated the average increase in HAZ among 12-23 months stunted children, within the same birth cohort, due to scaled-up nutrition interventions. For more details on the assumptions regarding the impact of interventions on linear growth and stunting, please see the LiST website (www.livessavedtool.org).

### Education attainment

For the transition between improved linear growth and highest grade attained, we relied on the pooled analysis from five major cohort studies in LMICs that linked early-childhood growth to educational attainment [12]. Assuming education improvements resulting from improved early-childhood growth are a linear function of HAZ, we applied the same number – 0.47 years of schooling per unit increase in HAZ at the age of 2 years among stunted children to the 156 LMICs included in LiST [12].

### Economic returns to schooling

For the transition between education and wage, we relied on a systematic review reporting data from 137 countries on percentages of wage increase per year of schooling, among which 132 countries were included in LiST [10]. For the remaining 24 countries in LiST with missing data, medians for each WHO region based on available data were applied.

### Net present value of lifetime earnings

To compute the net present value of lifetime earnings, we first referred to Wong et al. for the formula for calculating average annual wage [13]:

Wage = (GNI per capita × labor share of income)/labor force participation rate

Country-specific GNI per capita, converted in US dollar using Atlas methods, were retrieved from the World Bank database [14]. The labor share of income was defined as the part of national income allocated to wages and was assumed to be 50% for all countries. The first two terms provided an estimate of wage per capita. Since we wanted to estimate wage per worker, we applied the last factor – labor force participation rate, which was defined as percentage of workers among the total population. The World Bank reported percentage of workers among those who are 15 years or older, so we used the percentage of population ages 0-14 years old to estimate labor force participation rate as a percentage of the total population [14]. Country-specific labor force participation rates were available for most countries included in LiST. For countries with missing data on GNI per capita and labor force participation rate, the medians of corresponding income status countries were used. The labor share of income and labor force participation rate were held constant when calculating future wages.

We then used annual GNI per capita growth rate to project wages in future. The country-specific annual GNI per capita growth rates for 149 LMICs in LiST were available from the IIASA-SSP public database [15]. For the remaining 7 countries with missing data, medians for each WHO region based on available data were used. IIASA-SSP projects annual growth rates using five different models and reports the growth rates for every five years. The average of the projected annual growth rates across five models were applied to each country for the specific five-year period. We included the projected annual GNI per capita growth rates from 2020 to 2080 for each LMIC included in LiST.

Since additional wage earnings will accrue only once for each child as the birth cohort enter the labor market, we assumed the average working years is 44, starting at the age of 16 years and retiring at the age of 60 years. We also applied discounting rates to calculate the net present values of the future wages [16]. We adopted three commonly used discounting rates: 3%, 5%, and 10%. To estimate the total lifetime earnings gained per birth cohort, we used birth cohort size estimates from the United Nations Population Division’s World Population Prospects 2019 Revision [17].

### LiST projection

In LiST, users can model the scale-up of a wide range of interventions along the MNCH continuum of care to reduce the prevalence of stunting among children under five. LiST is embedded in the software package Spectrum, which also includes a demographic model. Users also have the option to directly change stunting prevalence. Details on the stunting in LiST are described elsewhere [11]. We modified stunting status in five countries of South Asia (Bangladesh, Bhutan, India, Nepal, and Pakistan) to reach the SDG target 2.2 by 2025 – the prevalence of stunting reduced by 40% from 2012 levels [4]. Assuming the prevalence of stunting among children under five reduces linearly from 2020 to 2025 and reaches the target, we created projections in LiST to estimate the additional years of schooling and present value of lifetime earnings for 6 birth cohorts of 2020-2025.

These projections are not mean to reflect achievable changes in stunting rates over a five-year period. Rather they do reflect the gains in schooling and lifetime earnings that would accrue if countries could reach the SDG targets for stunting.

## RESULTS

Based on our method, the median percent of wage increase per unit increase in HAZ among 156 LMICs included in LiST is 2.44%. The median percentage of wage increase per unit increase in HAZ by region are presented in **Table 1**. Country-specific associations among improved linear growth, education attainment and economic returns are available in Spectrum or LiST online (https://list.spectrumweb.org/).

**Table 1:** Median percentage of wage increase per unit increase in HAZ by region

Region	Median percentage of wage increase per unit increase in HAZ	Number of countries
Africa	2.4%	47
Americas	4.5%	30
Eastern-Mediterranean	2.4%	20
European	2.1%	29
South-East Asia	2.7%	11
Western-Pacific	3.5%	19
Overall	2.4%	156

HAZ – Height-for-age z-score

**Figure 2:**
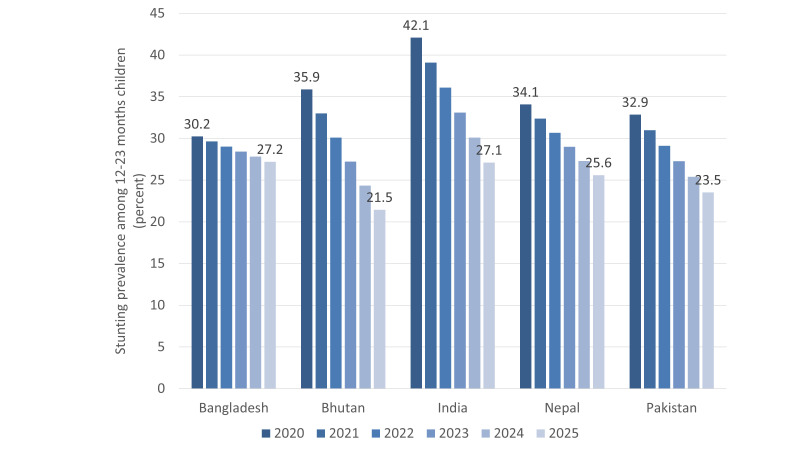
Estimated stunting prevalence among 12-23 months children in South Asia from 2020-2025 to reach Sustainable Development Goals.

If SDG target for stunting is reached in 2025, the prevalence of stunting among 12-23 months children will be 27.2%, 21.5%, 27.1%, 25.6%, and 23.5% in 2025 in Bangladesh, Bhutan, India, Nepal, and Pakistan, respectively. The prevalence of stunting is projected to reduce linearly from 2020 to 2025 (**Figure 2**).

Starting in 2020, if the stunting prevalence for children under five meets the SDG target by 2025 in South Asia, a total of 8563 additional years of schooling will be obtained due to improved linear growth among stunted children. India has the largest expected gains in schooling – an estimated 7367 additional years of education will be obtained by six birth cohorts. Additional years of schooling obtained per birth cohort by country are displayed in **Table 2**.

**Table 2:** Additional years of schooling gained per birth cohort due to improved linear growth (thousands of school years)

Countries	2020 birth cohort	2021 birth cohort	2022 birth cohort	2023 birth cohort	2024 birth cohort	2025 birth cohort	Total
Bangladesh	7.1	14	21	28	35	35	140
Bhutan	0.2	0.3	0.5	0.7	0.9	0.8	3.4
India	359	721	1092	1470	1865	1860	7367
Nepal	4	8	13	17	21	20	83
Pakistan	46	94	143	194	247	247	971
Total	416	837	1269	1709	2169	2163	8563

When using a 5% discount rate, the present value of additional lifetime earnings gained due to improved linear growth among stunted children in South Asia worth 64.888 million US dollars. The additional lifetime earnings in present value terms, at 3% and 10% discount rates are presented in **Table 3**

**Table 3:** Present value of additional lifetime earnings gained per birth cohort due to improved linear growth in South Asia* at different discount rate (US$ millions)

Discount rate	2020 birth cohort	2021 birth cohort	2022 birth cohort	2023 birth cohort	2024 birth cohort	2025 birth cohort	Total
3%	5750	11879	18 489	25 561	33 286	34 062	129 028
5%	2879	5956	9282	12 850	16 755	17 166	64 888
10%	677	1404	2195	3048	3986	4094	15 404

*Including Bangladesh, Bhutan, India, Nepal, and Pakistan.

The South Asia country with the greatest expected gains in lifetime earnings is India, with an estimated US$9.898 million gain per birth cohort in average in present value terms, at a 5% discount rate. Present value of additional lifetime earnings obtained per birth cohort due to improved linear growth at a 5% discount rate are displayed in **Table 4**. Present value of additional lifetime earnings obtained per birth cohort at alternative discount rates (3% and 10%) are available in Tables S1 and S2 in the **Online Supplementary Document**.

**Table 4:** Present value of additional lifetime earnings gained per birth cohort due to improved linear growth at 5% discount rate (US$ millions)

Countries	2020 birth cohort	2021 birth cohort	2022 birth cohort	2023 birth cohort	2024 birth cohort	2025 birth cohort	Total
Bangladesh	32	67	103	140	180	184	706
Bhutan	1	3	4	6	7	7	28
India	2640	5457	8501	11 760	15 329	15 702	59 390
Nepal	10	20	31	43	55	56	215
Pakistan	195	409	643	901	1183	1217	4549
Total	2879	5956	9282	12 850	16 755	17 166	64 888

**Figure 3:**
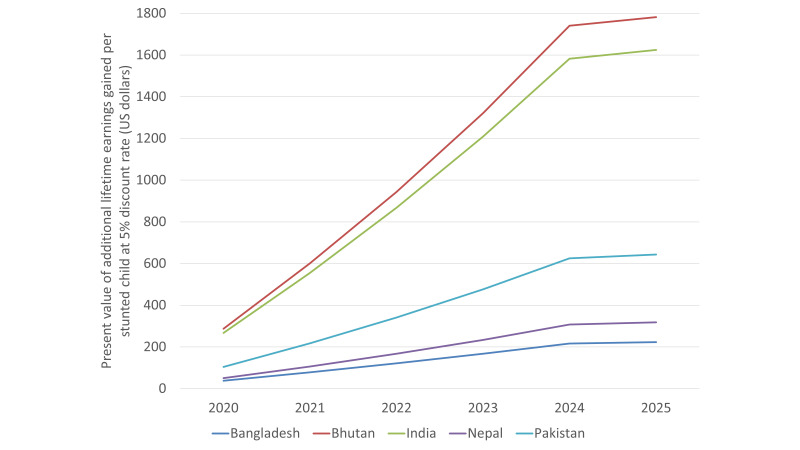
Present value of additional lifetime earnings gained per stunted child due to improved linear growth at 5% discount rate (US$).

**Table 5** reports present value of additional lifetime earnings gained per stunted child from 2020 to 2025 birth cohorts. Among the five South Asia countries, Bhutan has the largest gains in lifetime earnings per stunted child (**Figure 3**). For stunted children born between 2020 and 2025 in Bhutan, on average one stunted child is expected to gain US$1113 additional lifetime earnings due to improved linear growth in present value terms, at 5% discount rate. Present value of additional lifetime earnings obtained per stunted child at alternative discount rates (3% and 10%) are available in Tables S3 and S4 in the **Online Supplementary Document**.

**Table 5:** Present value of additional lifetime earnings gained per stunted child due to improved linear growth at 5% discount rate (US dollars)

Countries	2020	2021	2022	2023	2024	2025	Average
Bangladesh	38	78	121	168	217	223	141
Bhutan	287	601	943	1321	1740	1782	1113
India	267	555	868	1209	1582	1625	1018
Nepal	51	106	167	234	307	318	197
Pakistan	104	217	341	476	625	643	401

## DISCUSSION

With a 40% reduction in stunting prevalence from 2012 level by 2025, an estimated 8.6 million years of schooling will be obtained by six birth cohorts of 2020 to 2025 in South Asia. And the six birth cohorts will also gain an estimated 64.893 million USD dollars in present value term, at a 5% discount rate. India has the largest expected gains in years of schooling and lifetime earnings by birth cohorts. Bhutan has the greatest expected gains per stunted child.

There are two commonly used methods to estimate the association between reduction in stunting and subsequent economic outcomes. One is to measure the direct association based on long-term follow-up studies or prospective studies. There are two heavily referenced long-term follow-up studies: One was conducted in Guatemala and found a 46% increase in average wages for males who received the supplement as children [18]. The other was conducted in Jamaica and found a 25% increase in average earning for participants who received early childhood nutrition supplementation and psychosocial stimulation intervention [19]. Some prospective studies also looked at early life linear growth and economic consequences. They used various exposure measure, including height or height-for-age z score (HAZ) at different ages. There were also varied economic outcomes, ranging from physical work capacity, socioeconomic status, employment to income [20-22].

The second approach is based on calibrated estimates of returns to schooling, which is the method we used [10]. We decided to use the approach for the following reasons: 1) We want to include both additional years of schooling and LTE in the LiST model. Education attainment is an important outcome, especially among young women, given the strong association between maternal education and child survival. It was estimated that about 4.2 million deaths averted in children younger than 5 years can be attributed to the increase in women’s education between 1970 and 2009 in 175 countries [23]; 2) The evidence is sparse for direct association of reduction in stunting and wage; 3) The association is likely to vary by country evidenced by the two long-term follow up studies. We have chosen to use the data sets from a broader set of countries.

Our estimated median percentage of wage increase (2.44%) per unit increase in HAZ is lower than previous finding, where it was estimated that one unit increase in HAZ results in an 8% increase in annual income in Brazil and Guatemala [19]. The estimated lifetime productivity benefits are greatly impacted by discount rate given the long-lasting nature of the benefits and the fact that the increase in wage is experienced 16 years after the improvement in linear growth. Moving from a 5% to 3% discount rate doubles the benefits (**Table 3**).

One limitation of our approach is that we estimated the improved linear growth at the birth cohort-level for each country. We did not account for heterogeneity within countries. Although growth faltering affects a large fraction of the current child population in most LMICs, the cohort-level improvement will not be equally distributed to all stunted children.

In LiST, we store the historical data of GNI per capita from 2000 to 2019. We also store country-specific trends of annual GNI per capita growth rates calculated from IIASA-SSP public database. For labor force participation rate, we have one default country-specific value. Labor share of income is one default value for all countries. Users also have the option to modify one or more values of the indicators if they have better insight of the specific situation in a country. The outputs are displayed per annual birth cohorts. For present value of additional lifetime earnings, user can choose to display results for one of the three discount rates (3%,5%, or 10%).

Two other major benefits of nutrition interventions—avoided mortality and morbidity can also result in improved economic productivity [1,2]. Although currently there are no inputs to convert avoided mortality and morbidity into monetary values, these will be our next step to provide a complete set of the benefits of nutrition programming.

## CONCLUSIONS

Improved linear growth can produce both health-related and non-health-related benefits. Using the existing evidence, we added two outcomes – additional schooling and lifetime earnings obtained due to improved linear growth in the most recent version of LiST. LiST was already a useful tool to conduct cost-effectiveness analysis because we included all the health benefits of various interventions. With the addition of non-health benefit – lifetime earnings gained as output – together with LiST costing, users can now also conduct benefit-cost analysis. Cost-effectiveness analysis helps to determine how to best allocate health care resources, while benefit-cost analysis can be used to inform how to allocate resources both within and across sectors. Using both analyses will provide more comprehensive insights into the relative costs and benefits of nutrition interventions.

## Additional material


Online Supplementary Document
