# Supplementary material for: Estimating additional schooling and lifetime earning obtained from improved linear growth in low- and middle-income countries using the Lives Saved Tool (LiST)
Source: J Glob Health. 2022 Apr 2;12:08004. doi: 10.7189/jogh.12.08004 (PMC8974535; doi:10.7189/jogh.12.08004)
Supplement: Online Supplementary Document [file jogh-12-08004-s001.pdf]

Table S1. Present value of additional lifetime earnings gained per birth cohort due to improved linear growth at 3% discount rate (US$ millions)

Countries	2020 birth cohort	2021 birth cohort	2022 birth cohort	2023 birth cohort	2024 birth cohort	2025 birth cohort	Total
Bangladesh	65	134	206	281	360	367	1,414
Bhutan	2.6	5.2	8.1	11	14	15	56
India	5,269	10,874	16,916	23,369	30,422	31,123	117,973
Nepal	20	41	64	88	113	114	440
Pakistan	393	824	1,295	1,812	2,377	2,443	9,144
Total	5,750	11,879	18,489	25,561	33,286	34,062	129,028

Table S2. Present value of additional lifetime earnings gained per birth cohort due to improved linear growth at 10% discount rate (US$ millions)

Countries	2020 birth cohort	2021 birth cohort	2022 birth cohort	2023 birth cohort	2024 birth cohort	2025 birth cohort	Total
Bangladesh	8	15	24	33	42	43	165
Bhutan	0.31	0.64	1.0	1.4	1.8	1.8	6.9
India	622	1,289	2,014	2,795	3,654	3,752	14,126
Nepal	2.1	4.5	7.0	9.6	12	13	48
Pakistan	45	95	149	209	276	284	1,058
Total	677	1,404	2,195	3,048	3,986	4,094	15,404

Table S3. Present value of additional lifetime earnings gained per stunted child due to improved linear growth at 3% discount rate (US dollar)

Countries	2020	2021	2022	2023	2024	2025	Average
Bangladesh	76	157	244	336	434	447	282
Bhutan	568	1,186	1,860	2,600	3,420	3,498	2,189
India	532	1,106	1,727	2,402	3,140	3,220	2,021
Nepal	104	218	342	478	628	649	403
Pakistan	210	438	687	958	1,255	1,291	806

Table S4. Present value of additional lifetime earnings gained per stunted child due to improved linear growth at 10% discount rate (US dollar)

Countries	2020	2021	2022	2023	2024	2025	Average
Bangladesh	9	18	28	39	51	52	33
Bhutan	69	145	228	320	423	434	270
India	63	131	206	287	377	388	242
Nepal	11	24	37	52	69	72	44
Pakistan	24	50	79	111	145	150	93
